# Enoxacin adversely affects *Salmonella enterica* virulence and host pathogenesis through interference with type III secretion system type II (T3SS-II) and disruption of translocation of *Salmonella* Pathogenicity Island-2 (SPI2) effectors

**DOI:** 10.71150/jm.2410015

**Published:** 2025-02-27

**Authors:** El-Sayed Khafagy, Gamal A. Soliman, Maged S. Abdel-Kader, Mahmoud M. Bendary, Wael A. H. Hegazy, Momen Askoura

**Affiliations:** 1 Department of Pharmaceutics, College of Pharmacy, Prince Sattam bin Abdulaziz University, Al-kharj 11942, Saudi Arabia; 2 Department of Pharmacology, College of Pharmacy, Prince Sattam Bin Abdulaziz University, Al-Kharj 11942, Saudi Arabia; 3 Department of Pharmacology, College of Veterinary Medicine, Cairo University, Giza 12211, Egypt; 4 Department of Pharmacognosy, College of Pharmacy, Prince Sattam Bin Abdulaziz University, Al-Kharj 11942, Saudi Arabia; 5 Department of Pharmacognosy, Faculty of Pharmacy, Alexandria University, Alexandria 21215, Egypt; 6 Department of Microbiology and Immunology, Faculty of Pharmacy, Port Said University, Port Said 42511, Egypt; 7 Department of Microbiology and Immunology, Faculty of Pharmacy, Zagazig University, Zagazig 44519, Egypt; 8 Department of Pharmaceutical Sciences, Pharmacy Program, Oman College of Health Sciences, Muscat 113, Oman

**Keywords:** *Salmonella*, enoxacin, type three secretion system, anti-virulence

## Abstract

*Salmonella enterica* is a clinically significant oro-fecal pathogen that causes a wide variety of illnesses and can lead to epidemics. *S. enterica* expresses a lot of virulence factors that enhance its pathogenesis in host. For instance, *S. enterica* employs a type three secretion system (T3SS) to translocate a wide array of effector proteins that could change the surrounding niche ensuring suitable conditions for the thrive of *Salmonella* infection. Many antimicrobials have been recently introduced to overcome the annoying bacterial resistance to antibiotics. Enoxacin is member of the second-generation quinolones that possesses a considerable activity against *S. enterica*. The present study aimed to evaluate the effect of enoxacin at sub-minimum inhibitory concentration (sub-MIC) on *S. enterica* virulence capability and pathogenesis in host. Enoxacin at sub-MIC significantly diminished both *Salmonella* invasion and intracellular replication within the host cells. The observed inhibitory effect of enoxacin on *S. enterica* internalization could be attributed to its ability to interfere with translocation of the T3SS effector proteins. These results were further confirmed by the finding that enoxacin at sub-MIC down-regulated the expression of the genes encoding for T3SS-type II (T3SS-II). Moreover, enoxacin at sub-MIC lessened bacterial adhesion to abiotic surface and biofilm formation which indicates a potential anti-virulence activity. Importantly, in vivo results showed a significant ability of enoxacin to protect mice against *S. enterica* infection and decreased bacterial colonization within animal tissues. In nutshell, current findings shed light on an additional mechanism of enoxacin at sub-MIC by interfering with *Salmonella* intracellular replication. The outcomes presented herein could be further invested in conquering bacterial resistance and open the door for additional effective clinical applications.

## Introduction

Enoxacin, a broad-spectrum bactericidal fluoroquinolone antibiotic, acts by inhibiting bacterial DNA gyrase and topoisomerase IV that results in the disruption of bacterial DNA replication (Jałbrzykowska et al., 2022). Enoxacin has been efficiently prescribed for treating a wide range of bacterial infections, mainly urinary tract infections, prostatitis and gonorrhea (Alkhalil, 2024; Gutiérrez-Castrellón et al., 2015). Additionally, enoxacin has been effectively used to treat respiratory tract infections, including community-acquired pneumonia and acute bronchitis (Redgrave et al., 2014), bone and joint infections (Liu et al., 2014; Yao et al., 2021), skin and soft tissue infections (Kwak et al., 2017; Yao et al., 2021). Furthermore, enoxacin has been incorporated in the prophylaxis against bacterial infections in neutropenic patients, such as those undergoing chemotherapy for cancer (Yao et al., 2021). Enoxacin enhances RNA interference and promotes microRNA processing, in addition to generating free radicals. Notably, beyond its proapoptotic effects, induction of cell cycle arrest, and cytostatic properties, enoxacin also reduces cancer cell invasiveness (Jałbrzykowska et al., 2022). One of the most important applications of enoxacin is that it retains a considerable effectiveness against various Gram-negative bacteria, making it a potential alternative to ciprofloxacin for treating enteric fever, which is primarily caused by *S. enterica* serotypes Typhi and Paratyphi (Charles, 2017; Zehra et al., 2015).

*S. enterica* is a facultative intracellular Gram-negative bacterium which causes a range of gastrointestinal diseases expands from localized gastroenteritis to serious systematic fever known as enteric fever or typhoid (Hegazy and Hensel, 2012; Wain et al., 2015). *S. enterica* is the causative agent of fecal-oral transmitted diseases which can be categorized into typhoidal and non-typhoidal. However, while *Salmonella* serovars Typhi and Paratyphi A, B, and C give rise to enteric fever, the other serovars are classified as non-typhoidal (Buckle et al., 2012). Clinically, both types of infections are invasive and could lead to serious complications if not treated properly resulting in higher mortality rates (Harish and Menezes, 2011). Typhoidal *Salmonella* strains are human-specific pathogens responsible for enteric fever, commonly known as typhoid fever. In contrast, non-typhoidal strains can cause typhoid-like illness in other vertebrate animals but are primarily associated with localized gastroenteritis in humans (Buckle et al., 2012; Hung et al., 2017). While, typhoidal *Salmonella* causes serious systematic fever, the invasive non-typhoidal serovars such as Typhimurium or Enteritidis could cause bloodstream infections leading to serious illness (Askoura et al., 2021; Uche et al., 2017). Eventually, *Salmonella* infections pose significant health risks worldwide, particularly in Middle East, East Asia, and India (Rahman et al., 2014).

Pathogenicity islands (PAIs) are distinct genetic elements found within the genomes of pathogenic bacteria which contain clusters of genes contributing to the bacterium's ability to cause disease (Schmidt and Hensel, 2004). The genes that encode *Salmonella* virulence factors are clustered in *Salmonella* pathogenicity islands (SPIs). *Salmonella* species, including *S. enterica*, possess several SPIs that contribute to their virulence. The exact number of SPIs can vary among different strains and serovars of *Salmonella* (Marcus et al., 2000). Nonetheless, *S. enterica* harbors a total of seventeen SPIs (Vernikos and Parkhill, 2006), with SPI1 and SPI2 being the foremost and extensively researched among them (Hegazy and Hensel, 2012; Hegazy et al., 2012).

Bacteria recruit various types of secretion systems (SSs) to transport proteins and other molecules across their cell membranes and into their external environment. These SSs play crucial roles in bacterial physiology, virulence, and interactions with surrounding environments (Alandiyjany et al., 2022; Troman and Collinson, 2021). Among the most characterized SSs, type III secretion system (T3SS) is a complex molecular machinery found in some Gram-negative bacteria that enables them to inject virulence factors directly into the cytoplasm of host cells. T3SS resembles a molecular syringe, allowing bacteria to deliver proteins directly into the cytoplasm of host cells, where they manipulate cellular processes to the bacterium's advantage (Cornelis, 2006; Hegazy et al., 2012). *Salmonella* is renowned for its ability to produce a T3SS that play crucial roles during various infection phases (Kuhle and Hensel, 2004). Two key SPIs, SPI1 and SPI2, encode T3SS and are responsible for the production of diverse bacterial effectors (Cornelis, 2006; Hegazy et al., 2022). SPI1 is essential for bacterial invasion, whereas SPI2 is critical for intracellular survival within human immune cells as antigen presenting cells including macrophages and dendritic cells that facilitates bacterial proliferation and establishment of systemic infection in the host (Elfaky et al., 2022; Hegazy and Abbas, 2017; Thabit et al., 2022). Following ingestion of *S. enterica*-contaminated food and/or drink; it reaches the gastrointestinal tract and uptaken by gate cells (M cells) in the Peyer's patches lining the intestine (Askoura and Hegazy, 2020; Kamaruzzaman et al., 2017; Thabit et al., 2022). In the intestine, *Salmonella* cells are then engulfed within the macrophages phagosomes which are known as *Salmonella*-containing vacuole (SCVs) where *Salmonella* can survive by secreting SPI2 effector proteins that prevent the fusion of phagosome with lysosomes, thereby evading lysosomal killing (Cornelis, 2006; Elfaky et al., 2022).

**Fig. 1.**
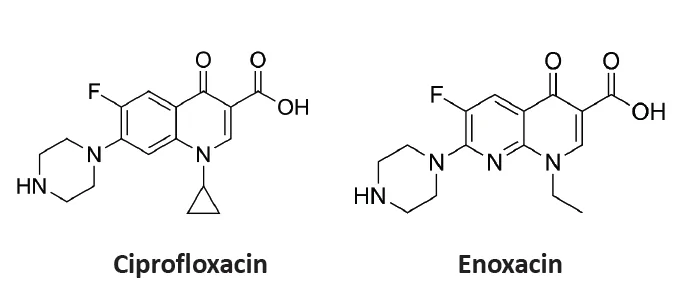
Chemical structure similarity between enoxacin and ciprofloxacin. Enoxacin: 1-ethyl-6-fluoro-4-oxo-7-piperazin-1-yl-1,8-naphthyridine-3-carboxylic acid; ciprofloxacin: 1-cyclopropyl-6-fluoro-4-oxo-7-piperazin-1-ylquinoline-3-carboxylic acid.

In spite of the documented resistance, the fluorinated quinolones, particularly the second-generation members such as ciprofloxacin, are still effectively employed in treating of systemic salmonellosis because of their bactericidal properties and heightened efficacy within host cells (Easmon et al., 1986; Rahman et al., 2014). Intriguingly, ciprofloxacin at sub-MIC showed a significant ability to interfere with the SPI2 functionality, diminishing the expression of SPI2 encoding genes and lessened the translocation of the SPI2 effectors leading to a significant defect in *S. enterica* ability to survive and replicate intracellularly within the host cells (Askoura and Hegazy, 2020). Enoxacin, another member of second-generation quinolones, shares ciprofloxacin the fluorinated quinolone moiety (Fig. 1). Enoxacin has been shown to interfere intracellularly with RNA in cancer cells that results in the promotion of microRNA (Jałbrzykowska et al., 2022; Shaw and Gullerova, 2021), and the production of intracellular free radicals (Hussen et al., 2023). Bearing in mind these findings, it is hypothesized that enoxacin could display an additional antibacterial mechanism of action against intracellular bacteria. The current study aims to endorse the enoxacin interference with *S. enterica* T3SS, and its ability to protect mice in vivo against bacterial infection.

## Materials and Methods

### Determination of the effect of enoxacin sub-minimum inhibitory concentration (sub-MIC) on *S. enterica* growth

*S. enterica* serovar Typhimurium NCTC 12023 was used in this study. Enoxacin (Cas no.74011-58-8) was purchased from Santa Cruz Biotechnology (USA). The broth microdilution method was employed to determine the MIC of enoxacin following the Clinical Laboratory and Standards Institute (CLSI, 2015) guidelines with minor modifications (Abdulaal et al., 2024). The influence of enoxacin at sub-MIC on bacterial growth was assessed spectrophotometrically. The optical densities of *S. enterica* grown in Luria-Bertani (LB) broth with and without enoxacin at sub-MIC were measured at different time intervals (Nazeih et al., 2023).

### Bacterial adhesion and biofilm assay

Fresh cultures of *S. enterica* treated with enoxacin at sub-MIC were diluted with fresh Tryptic Soy Broth (TSB) and attuned to a cell density of 1 × 10^6^ CFU/ml (OD_600_= 0.4) for adhesion and biofilm assay (Vesterlund et al., 2005). To increase the adhesion and biofilm formation (Askoura et al., 2021), the bacterial suspensions of *S. enterica* were cultured in the presence of N-hexanoyl-DL-homoserine lactone (0.001 µM) and incubated at 37°C for 1 h or 24 h for evaluation the bacterial adhesion, or biofilm formation (Askoura et al., 2021; Stepanovic et al., 2000). Plates were washed to remove non-adherent cells, adhered cells were fixed with for 30 min at 65°C and stained with 0.1% crystal violet for 25 min. Excess crystal violet was removed and adhered dye was extracted with methanol that was finally measured spectrophotometrically at 590 nm (Hegazy and Abbas, 2017; Koshak et al., 2024; Vesterlund et al., 2005).

### Invasion and intracellular replication assay

Gentamicin protection assay was used to assess the impact of enoxacin at sub-MIC on *S. enterica* invasion and intracellular replication as previously described (Askoura et al., 2021; Askoura and Hegazy, 2020; Hegazy et al., 2012). Briefly, 24-wells polystyrene plates were seeded with HeLa cells or RAW264.7 macrophages at cell density of 5 × 10^5^ and 2 × 10^5^ cells/well for invasion and intracellular replication assay, respectively. Overnight *S. enterica* cultures provided or not with enoxacin at sub-MIC were grown for 3 to 4 h at 37°C. Bacterial inoculum (1 × 10^5^ bacterial cells/well) was mixed with eukaryotic cells (HeLa cells or macrophages) at a multiplicity of infection (MOI) of 1 in Dulbecco’s modified Eagle’s medium (DMEM) in the presence of 0.001 µM N-hexanoyl-DL-homoserine lactone and incubated for 25 min. Non-internalized bacterial cells were washed out using phosphate buffer saline (PBS), while the adhered bacterial cells were killed by gentamicin (100 µg/ml) for 1 h. In order to evaluate bacterial invasion, HeLa cells were lysed using triton X-100 (0.1%) for 15 min at room temperature. The initial inoculum and intracellular bacteria were viably counted and the percentage of invading bacteria (bacterial count at 1 h post-infection/initial bacterial count × 100) was calculated. For the assay of bacterial intracellular replication, *Salmonella*-infected macrophages were lysed with 0.1% triton X-100 at 2 and 16 h post-infection exactly as described above. Next, the bacterial inoculum and intracellular bacteria were viably counted and the phagocytosed bacterial cells numbers comparative to uptaken cells (bacterial count at 2 h post-infection/initial bacterial count × 100) and x-fold intracellular replication (bacterial count at 16 h relative to bacterial count at 2 h) was calculated. The results are expressed as the means ± standard errors and the difference was considered significant at a *P* value of < 0.05 using ANOVA test.

### Quantification of the translocation of T3SS effectors

The translocation of SPI2 effector SseJ was quantified to assess the influence of enoxacin on *S. enterica* ability translocate SPI2-T3SS within host cells. The constructed plasmid pWsk29 P*_sseJ_sseJ*::hSurvivin encodes the hemagglutinin (HA) tagged SPI2 effector protein SseJ (Askoura et al., 2021; Hegazy et al., 2012) was transformed into *S. enterica* by electroporation. *S. enterica* harboring the plasmid was allowed to grow in the presence or absence of enoxacin at sub-MIC. Then bacteria were used to infect HeLa cells or RAW264.7 macrophages in the presence of 0.001 µM N-hexanoyl-DL-homoserine lactone at MOI of 100 (Askoura et al., 2021; Askoura and Hegazy, 2020; Hegazy et al., 2012). Infected cells were immune stained to assess the translocated effector proteins after 16 h using *Salmonella* LPS [rabbit anti-*Salmonella* O 1,4,5 (Difco, BD)] and the HA epitope tag (Roche, Switzerland). Secondary antibodies anti-rabbit tagged with GFP (green fluorescent protein) was used to stain *Salmonella* cells (Abcam; USA), the translocated SPI2 effector was stained with Cyanine5 (Cy5) dye (Invitrogen, USA) and diamidino-2-phenylindole dye (DAPI; Thermo Fisher Scientific, USA) was used to stain macrophages (Askoura et al., 2021; Hegazy et al., 2012; Xu et al., 2014). Leica laser-scanning confocal microscope was used analyze the translocation of SPI2 effector SseJ and images were captured. The fluorescence signal intensities of HA-tagged SseJ within infected cells were quantified using J-image program.

### RT-qPCR analysis

*S. enterica* was grown in SPI-2-inducing minimal phosphate-carbon-nitrogen medium (PCN-P, pH 5.8) to enhance the SPI-2 effector expression in the presence or absence of enoxacin (Deiwick et al., 1999). Both untreated and treated *S. enterica* with sub-MIC of enoxacin, were collected by centrifugation. Bacterial RNA was extracted using RNAeasy Mini Kit (Qiagen, Germany), quantified using NanoDrop ND-1000 spectrophotometer and Kept at -80°C until use (Elfaky et al., 2024; Hegazy, 2015). The expression of different genes encoding for SPI2-effectors (*ssaJ*, *ssaV*, *steC*, *ssrB*, *sseJ*, *sifA*, *sifB*, *sseL*, *sscA*, *ssaE*, *sseF*, and *pipB*) in the presence of enoxacin was assessed using RT-qPCR. High-capacity cDNA reverse transcriptase kit (Applied Biosystem, USA) was used to obtain cDNA which was amplified in a multi-well plate using Step One instrument (Applied Biosystem, USA) using the Syber Green I PCR Master Kit (Fermentas, USA). Specific PCR amplification was verified using both agarose gel electrophoresis and melting curve analysis of the products following the manufacturer's recommendations (Elfaky et al., 2023). Comparative gene expression was calculated using the 2^−∆∆CT^ method. The used primers were previously listed, and the expression was normalized to the housekeeping gene *gyrB* (Askoura and Hegazy, 2020).

### In vivo protection against *S. enterica* pathogenesis using mice infection model

Three-week-old albino mice received intraperitoneal injections (I.P) of *S. enterica* treated or not with enoxacin at sub-MIC to evaluate its inhibitory effect on *S. enterica* pathogenesis (Badr-Eldin et al., 2024; Bendary et al., 2024). The experiment included four animal groups; each contains five mice. The first two groups, serving as negative controls, were left uninfected or injected with sterile PBS. Mice in the third group received *S. enterica* (1 × 10^6^ CFU/ml) treated with sub-MIC of enoxacin (2.5 µg/ml). Mice in last group were injected with *S. enterica* untreated with enoxacin and served as a positive control. Mice survival was monitored over 5 days and plotted using Kaplan-Meier method and the statistical analysis was verified using Log-rank test. Furthermore, the mice kidney and liver were isolated, homogenized and viable bacteria were counted. The homogenized organ tissues were transferred to sterile PBS, serially diluted and plated on *Salmonella Shigella* (SS) agar plates. The bacterial counts were assessed and expressed as colony-forming units (CFU) per gram of tissue. One-way analysis of variance (ANOVA) test was used to determine the statistical significance.

### Statistics

The presented data are the means ± standard error compared to untreated controls. Unless stated otherwise, significance was assessed using the student's *t*-test (*p* < 0.05).

## Results

### Enoxacin at sub-MIC diminished *S. enterica* adhesion to abiotic surface and reduced biofilm formation

**Fig. 2.**
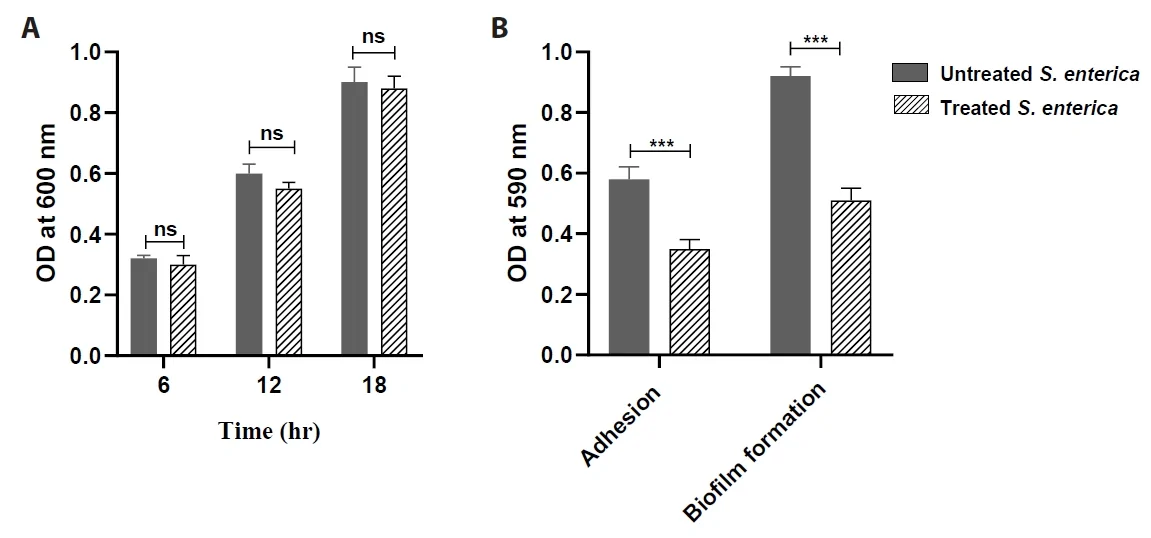
Characterization of the influence of enoxacin at sub-MIC on *S. enterica* growth and virulence capability (A) Enoxacin at sub-MIC has no effect on bacterial growth. (B) Enoxacin significantly reduced *S. enterica* adhesion to abiotic surface and biofilm formation. Enoxacin at sub-MIC decreased the adhesion to abiotic surface and biofilm formation by 40% and 45%, respectively in comparison to untreated control (ns: non-significant, ^*^P > 0.05, ^**^P < 0.01, ^***^P < 0.001).

The minimum inhibitory concentarion (MIC) of tnoxacin that inhibited *S. enterica* growth was determined and found to be 1 µg/ml. To ensure the enoxacin's anti-biofilm effect excluding any influence on bacterial growth, *S. enterica* growth was assessed spectrophotometrically by measuring the optical density of growing bacteria in presence and absence of enoxacin at 1/4 MIC (0.25 µg/ml) (Fig. 2A). There was no effect of enoxacin at sub-MIC on bacterial growth. The crystal violet method was used to quantify both the adhering bacterial cells to abiotic surface as well as biofilm forming capacity after 1 and 24 h, respectively. The optical density of the crystal violet significantly reduced when *S. enterica* were treated with enoxacin at sub-MIC (0.025 µg/ml) (Fig. 2B). This indicates that enoxacin at sub-MIC significantly decreased the bacterial adhesion to abiotic surface and biofilm formation by 40% and 45%, respectively.

### Enoxacin at sub-MIC reduced *S. enterica* invasion and intracellular replication

**Fig. 3.**
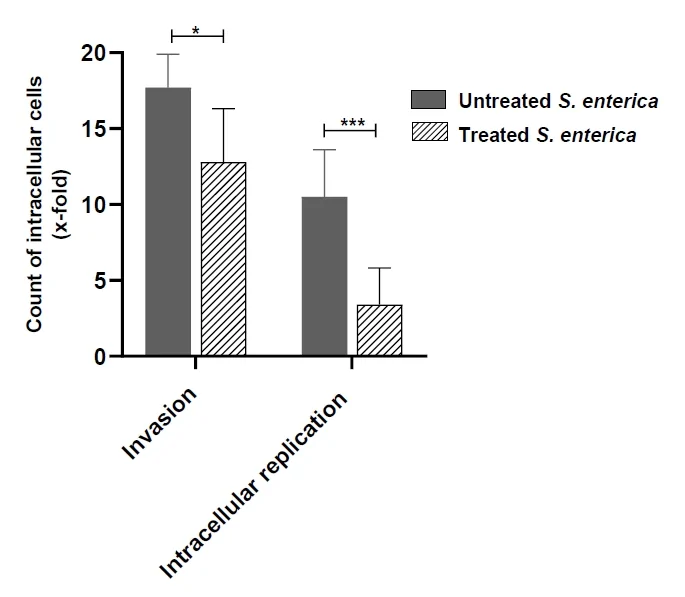
Enoxacin significantly reduced *S. enterica* invasion and intracellular replication. Enoxacin at sub-MIC significantly decreased the bacterial invasion and intracellular replication by 72.5 and 32.5% respectively, respectively in comparison to untreated control (^*^P > 0.05, ^**^P < 0.01, ^***^P < 0.001).

Gentamicin assay was used to evaluate the influence of enoxacin at sub-MIC (0.25 µg/ml) on *S. enterica* invasion into HeLa cells and intracellular replication within RAW264.7 macrophages. Enoxacin at sub-MIC significantly reduced both *S. enterica* invasion and intracellular replication by 72.5 and 32.5%, respectively (Fig. 3).

### Enoxacin interfered with the translocation of SPI2-effector proteins

**Fig. 4.**
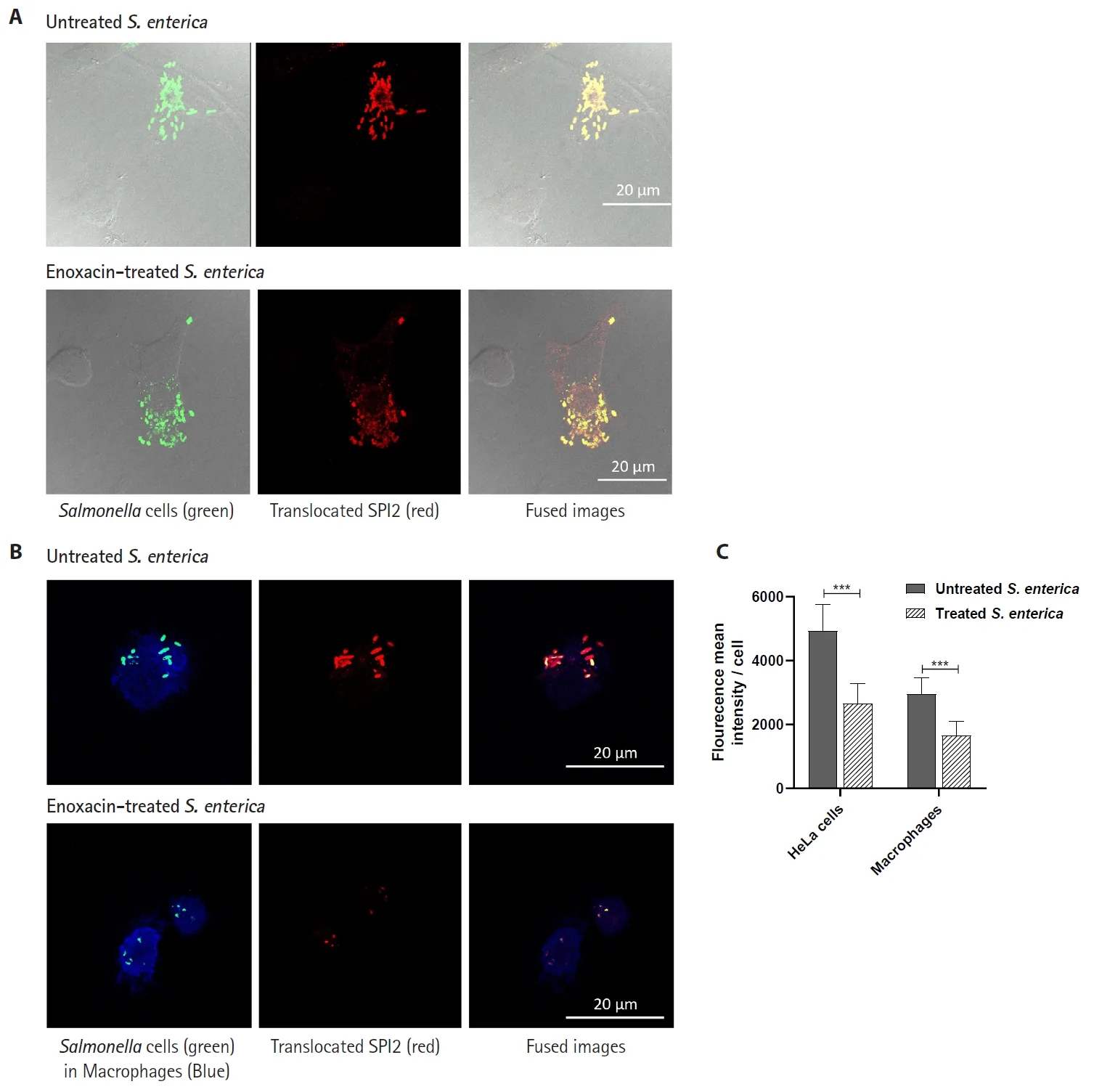
Enoxacin significantly interfered with the translocation of *S. enterica* SPI2-effectors. Infected HeLa cells (A) or macrophages (B) with *S. enterica* treated or not with enoxacin at sub-MIC were immune stained. Translocated HA-tagged Ssej was secondary stained with cy5 (red), while the bacterial cells were green stained (using GFP-secondary antibody), and the DAPI was used as counter stain for macrophages. (C) The intensity of the red fluorescence (translocated protein) was quantified using J-image program in at least 25 cells. Enoxacin significantly decreased the translocation of the SPI2 effector.

The translocation of the *S. enterica* SPI2 effector protein (HA-tagged SseJ) was quantified in the presence and absence of enoxacin at sub-MIC levels to evaluate enoxacin effect on T3SS Type 2. The fluorescence of the translocated HA-tagged SseJ was quantified in *S. enterica* treated with enoxacin at sub-MIC (0.25 µg/ml) and compared to that in untreated bacteria (Fig. 4). The fluorescence intensity of the tagged SPI2 effector was significantly reduced in the presence enoxacin at sub-MIC, which indicates its interference with the translocation of SPI2 effector proteins.

### Enoxacin markedly reduced the expression of genes encode SPI2 effectors

**Fig. 5.**
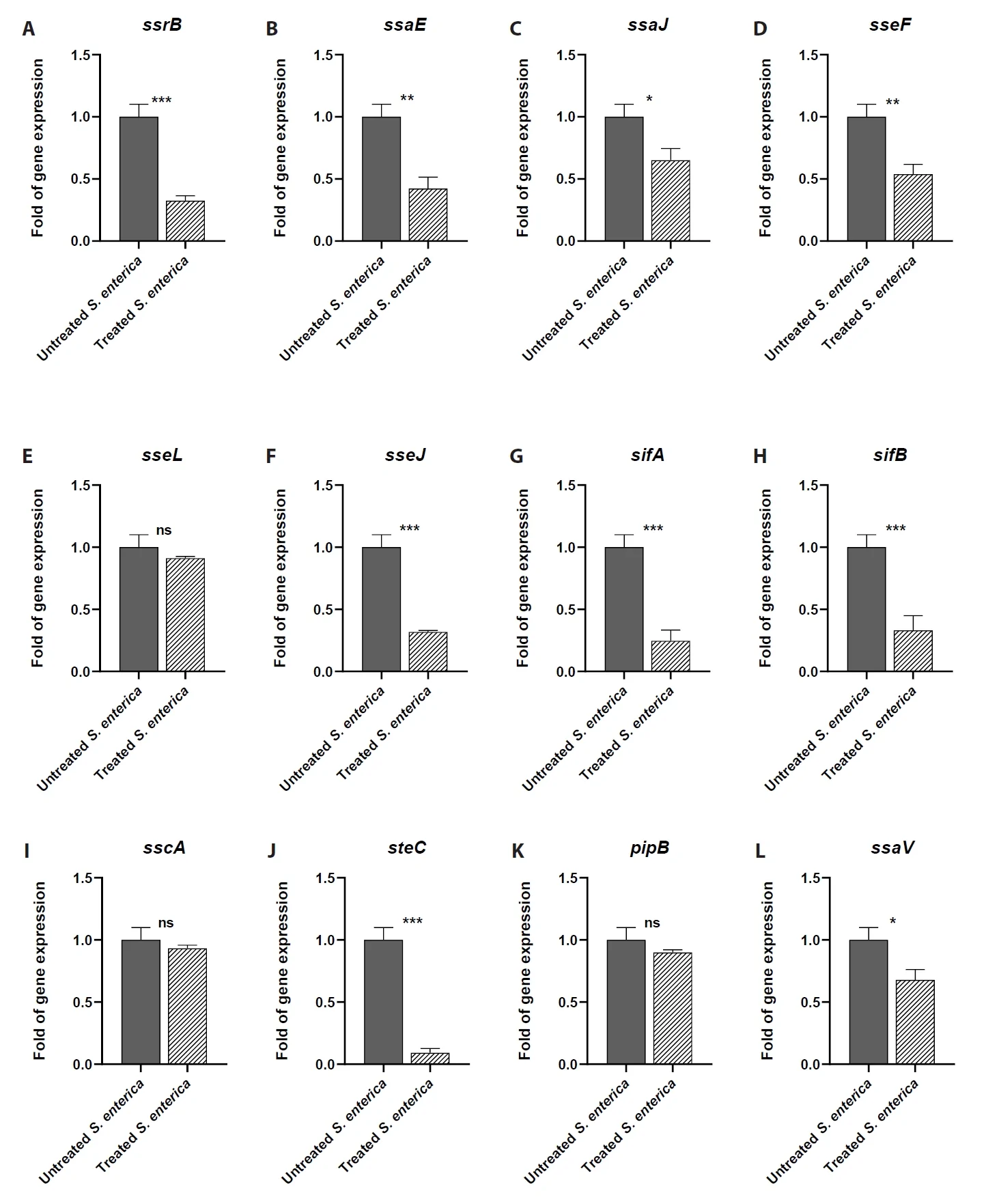
Enoxacin downregulated the expression of SPI2 genes. The expression of SPI2 genes were quantified and normalized to *gyrB*. Enoxacin at sub-MIC significantly downregulated the expression of all tested genes except *sseL*, *sscA* and *pipB* (ns: non-significant, *P* > 0.05, ^***^P < 0.001, ^**^P < 0.01, ^*^P < 0001).

The expression level of SPI-2 encoding genes was quantified using RT-qPCR in *S. enterica* treated or not with enoxacin at sub-MIC (0.25 µg/ml) (Fig. 5). Enoxacin significantly downregulated the expression of all tested SPI2 genes except *sseL*, *sscA*, and *pipB*. The down-regulation effect of enoxacin varied from 5-fold as in case of *steC* genes and 3-fold in genes *ssrB*, *sseJ*, *sifA*, and *sifB* to 2-fold in genes *ssaE* and *sseF*, while the least reduction in expression was observed in *ssaJ* and *ssaV*.

### Enoxacin alleviated *S. enterica* pathogenesis in mice

**Fig. 6.**
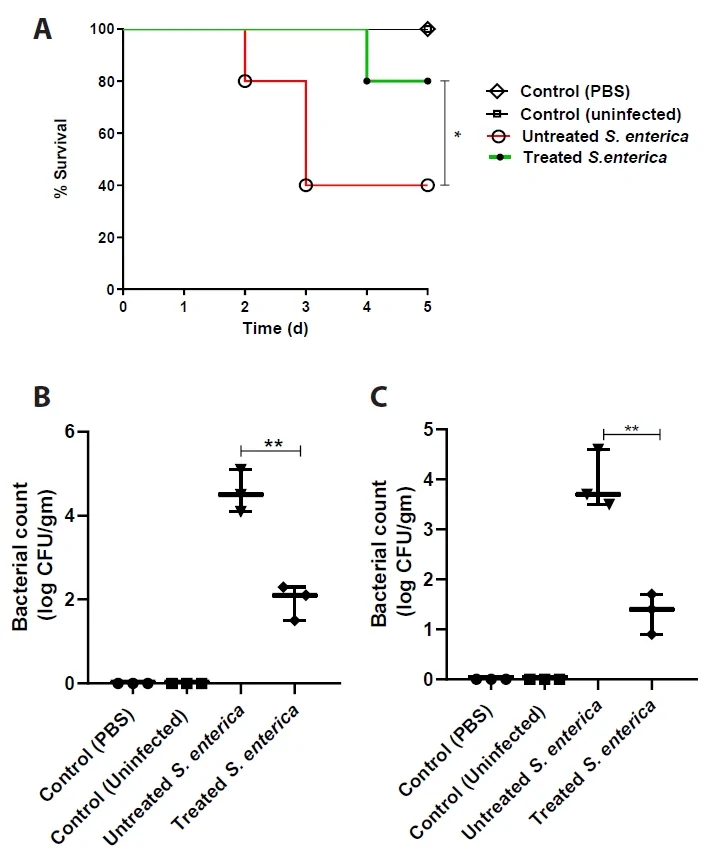
Enoxacin diminished *S. enterica* ability to colonize and establish infection in mice. (A) Mice survival was monitored in each group daily for 5 days and plotted using Kaplan-Meier survival curve. There were no deaths in negative control groups. Enoxacin at sub-MIC significantly (*P* = 0.0394) reduced mortality rate. Bacterial loads in (B) liver and (C) kidney tissues were determined. Colonizing bacteria in organs isolated from mice injected with enoxacin-treated *S. enterica* were significantly reduced compared with untreated *S. enterica*-injected mice (^**^P < 0.01, ^***^P < 0001).

In compliance with the interference capability of enoxacin with T3SS-type2 either by down-regulation of its encoding genes or interference with the translocation of its effectors, enoxacin significantly decreased *S. enterica* pathogenesis in mice. All mice survived in groups that were uninfected and PBS-injected (negative control groups). However, three deaths in mice out of five were reported in the mice injected with untreated *S. enterica*, while one death was recorded in the mice injected with *S. enterica* treated with enoxacin at sub-inhibitory concentration (0.25 µg/ml) (Fig. 6A). These results clearly indicate that enoxacin at sub-MIC reduced mice deaths by 66% (*P = 0.0394). Additionally, *S. enterica* load in isolated mice kidney and liver were determined for all mice groups. The results revealed a significant reduction in bacterial counts in organs isolated from enoxacin-treated mice as compared to untreated mice. This indicates a remarkable capability of enoxacin to diminish *S. enterica* colonization in host (Fig. 6B and C).

## Discussion

Clinically, typhoidal and non-typhoidal *S. enterica* are among the important pathogens that could easily spread via contaminated food and/or water causing endemics and epidemics (Crump et al., 2015). The development of bacterial resistance poses an imminent threat, particularly in the context of severe epidemic infections like the oro-fecal transmitted enteric fever caused by *S. enterica* (Tadesse et al., 2018). That requires the developing of new approaches to conquer bacterial resistance and deeply investigate the possible additional antibacterial effects of known antibiotics as well as new antimicrobial candidates (Agha et al., 2016; Khayat et al., 2022, 2023). In this study, it was aimed to evaluate the anti-virulence potential of enoxacin at sub-inhibitory concentrations against *S. enterica* serovar Typhimurium.

First, to ensure that enoxacin’s interference with the T3SS and its anti-virulence activities are due solely to enoxacin and not to any influence on *S. enterica* growth or viability, it was necessary to eliminate any influence of enoxacin on the growth of bacteria (Almalki et al., 2022; Alotaibi et al., 2023). The optical densities of growing bacteria in absence and presence of enoxacin at sub-inhibitory concentration were measured. There was no significant difference between enoxacin-treated and untreated cultures. This indicates that enoxacin has no influence on *Salmonella* growth at sub-MIC and therefore all tests were conducted at the same sub-inhibitory concentration of enoxacin (1/4 MIC; 0.25 µg/ml).

The injectosome of *S. enterica* T3SS is extensively studied for its crucial role in pathogenesis and infection establishment. *S. enterica* initiates its infection in host cells by the expression of T3SS-type I (T3SS-I) that is encoded by SPI1 genes (Kuhle and Hensel, 2004; Marcus et al., 2000). Following *S. enterica* adhesion, bacteria release SPI1-effectors into the cytoplasm of invaded host cells which results in deformation of the cellular G-proteins and facilitates bacterial cells invasion into the host cells (Deiwick et al., 1999; Kuhle and Hensel, 2004). The host cells could engulf invading salmonella cells into the SCV in order to facilitate their killing either by oxidizing or non-oxidizing pathways (Yin et al., 2017). However, *S. enterica* T3SS-type II (T3SS-II) that is encoded by SPI2 starts its crucial function. T3SS-II translocate a diverse array of proteins with different functions to ensure not only *Salmonella* survival in SCV, but also facilitate its thrive and proliferation within SCV (Cornelis, 2006; Patel and Galan, 2005). In this context, the interference with *Salmonella* T3SS both types I and II could result in diminishing the bacterial invasion and intracellular replication, respectively (Alandiyjany et al., 2022; Thabit et al., 2022). Intriguingly, our findings showed that enoxacin at sub-MIC significantly diminished *S. enterica* invasion into HeLa cells as well as intracellular replication within human macrophages. That indicates the potential enoxacin interference with the T3SS.

Taking into consideration the outmost importance of T3SS-II for *S. enterica* host pathogenesis and intracellular survival within host cells (Askoura and Hegazy, 2020; Hegazy and Hensel, 2012), the translocation of SPI2 effectors was evaluated in the presence of sub-MIC of enoxacin. For this purpose, a plasmid harboring HA tagged SPI2 effector SseJ (SseJ::HA) (Askoura et al., 2021; Hegazy et al., 2012) was transformed to *S. enterica* that then allowed to grow in the absence or presence of enoxacin at sub-inhibitory concentration. The fluorescence of translocated tagged SPI2 effector was greatly decreased in the cytoplasm of both infected HeLa cells and macrophages. This indicates the higher efficiency of enoxacin to adversely affect the translocation of SPI2 effectors and lessening of *S. enterica* ability to internalize the host cells which explains the significant reduction in bacterial invasion as well as intracellular replication.

There are more than thirty genes that encode the T3SS-II apparatus (Ssa), chaperones (Ssc) and effectors (Sse) in addition to a separate operon encodes the regulatory secretion system SsrAB (Hegazy and Hensel, 2012; Kuhle and Hensel, 2004). T3SS-II translocate diverse proteins "effectors" into the cytoplasm of the host cells, that secures the nutrition and thrive of the *Salmonella* cells in SCV (Cornelis, 2006; Holzer and Hensel, 2012). The interference of enoxacin with effectors translocation was further confirmed by RT-qPCR in order to characterize its effect on the expression of SPI2 genes which play a significant role in *Salmonella* host virulence and pathogenesis (Gerlach and Hensel, 2007). Interestingly, current results show that enoxacin down-regulated all the SPI2 genes at sub-MIC. For instance, enoxacin down-regulated *ssrB* that encodes the regulatory system as well as *ssaE* and *ssaJ* that encode for the structural proteins of T3SS apparatus. Additionally, both *sseJ* and *sseF* that encode SPI2 effectors were significantly down-regulated following *Salmonella* exposure to sub-MIC of enoxacin.

*Salmonella* launches its own intracellular replicative niche and establishes a dynamic network of *Salmonella*-induced filaments (SIFs). SIFs originate from SCV, extend throughout infected host cells connecting separate SCVs that attain a vital role in the intracellular survival of *Salmonella* (Knuff and Finlay, 2017). The biogenesis of SIFs is contingent on the activity of *sseF* that in turn enhances the expression of other two effectors SifA and SifB which are essential for microtubule bundling and SIF formation (Kuhle and Hensel, 2004). Enoxacin significantly down-regulated *sseF*, *sifA*, and *sifB* that encode for essential effectors in order to maintain SCV integrity (Gerlach and Hensel, 2007; Kuhle and Hensel, 2004). PipB is involved in the dynamics of the SCV within host cells. PipB assists, in cooperation with SifB, in the trafficking of SCV along microtubules, which is critical for maintaining of SCV integrity and positioning within the host cells (Knodler et al., 2002, 2003). While enoxacin repressed *sifA* and *sifB* expression, it did not show a marked effect on *pipB*. Chaperone proteins are specialized proteins that assist in the proper folding, assembly, and maintenance of other proteins within the cell, some of SPI2 effectors require chaperon protein as SscA (Cornelis, 2006, Gerlach and Hensel, 2007). Ssav is essential for secretion of many effectors necessary for *Salmonella* survival within the host cells, and its deletion significantly decrease both intracellular survival and host virulence (Askoura et al., 2021; Hegazy and Abbas, 2017; Yu et al., 2018). Current results showed the significant down-regulation of *ssaV*, but not on *sscA* following bacterial treatment with enoxacin. The most observed down-regulation was observed to *steC* which encodes for a multifunctional effector protein that plays a pivotal role in *Salmonella* pathogenesis by manipulating the host cell actin cytoskeleton, modulating signaling pathways, and supporting intracellular survival and replication. SteC is crucial for the bacterium's ability to create a conducive environment within host cells and to enhance its virulence (Heggie et al., 2021; Poh et al., 2008). Enoxacin at sub-MIC reduced the expression of T3SS-II encoding genes emphasizes its adverse effect on the translocation of SPI2 effectors and diminished *Salmonella* intracellular replication.

Bacterial adhesion and biofilm formation are critical processes in *Salmonella* lifecycle and host virulence (Askoura et al., 2021; Elfaky et al., 2022; Hegazy and Abbas, 2017). The process of biofilm formation involves several distinct stages that ends by the development of a complex, structured community of bacteria (Elfaky et al., 2023; Rajab and Hegazy, 2023). Formation of biofilms in chronic wounds impede healing and contribute to prolonged infection that in turn enhance the development of resistance to antibiotics. Therefore, eradicating biofilms is a crucial goal for effective treatment (Khayat et al., 2023; Lila et al., 2023; Nazeih et al., 2023). Quorum sensing (QS) is a fundamental communication mechanism that enables bacteria to coordinate collective behaviors essential for survival and pathogenicity. QS systems control the production of diverse virulence factors and biofilm formation (Cavalu et al., 2022; Khayat et al., 2022; Thabit et al., 2022). Present findings demonstrate the significant ability of enoxacin at sub-MIC to diminish both bacterial adhesion and biofilm formation. These findings suggest that enoxacin may possess anti- anti-QS activity, potentially explaining its additional anti-virulence potential. Further detailed investigations are needed to explore the anti-QS properties of enoxacin.

In line with the current results, the bactericidal doses of antibiotics could kill or inhibit the growth of targeted bacteria, while at sub-inhibitory concentration, they could induce physiological changes in these bacteria (Linares et al., 2006). These changes could affect a wide array of processes in the bacteria and lead to fitness modifications that influence bacterial thrive in different environments (Andersson and Hughes, 2014). Exposure to antibiotics at sub-inhibitory concentrations has been shown to modulate the expression of virulence-related processes, including QS, host-cell adherence, and bacterial motility (Molina-Quiroz et al., 2015). Furthermore, in vivo evidence showed the ability of fluoroquinolones such as ciprofloxacin at sub-inhibitory concentration to lessen *S. enterica* pathogenesis (Askoura and Hegazy, 2020). This is in compliance with the current results which disclosed the significant ability of enoxacin at sub-inhibitory concentration to diminish *Salmonella* virulence capability and host pathogenesis. In vivo findings are in alignment with the in vitro results which emphasize the interference of enoxacin with *Salmonella* invasion of host cells and consequently a decrease bacterial colonization of the host tissues

In conclusion, the present study clearly shows the interference ability of enoxacin at sub-MIC with both invasion and intracellular survival capabilities of *Salmonella* within the host cells. These effects could be attributed to enoxacin interference with T3SS-II. Enoxacin diminished the translocation of SPI2 effectors and reduced the expression of their encoding genes. These findings uncover that enoxacin exhibits an additional effective intracellular activity against *Salmonella* through disrupting of bacterial survival within host cells. These results are significant in expanding our understanding of the various mechanisms by which enoxacin and other quinolones could control bacterial infections. This approach could be beneficial in overcoming bacterial resistance to commonly used antibiotics. Furthermore, these observations pave the way to investigate and explore the anti-virulence activity of antibiotics and drugs that share similar chemical moieties.

## Data Availability

All the data are provided within this published article.

## References

[b1-jm-2410015] Abdulaal WH, Alhakamy NA, Asseri AH, Radwan MF, Ibrahim TS (2024). Redirecting pantoprazole as a metallo-beta-lactamase inhibitor in carbapenem-resistant *Klebsiella pneumoniae*. Front Pharmacol.

[b2-jm-2410015] Agha KA, Abo-Dya NE, Ibrahim TS, Abdel-Aal EH, Hegazy WA (2016). Benzotriazole-mediated synthesis and antibacterial activity of novel *N*-acylcephalexins. Sci Pharm.

[b3-jm-2410015] Alandiyjany MN, Abdelaziz AS, Abdelfattah-Hassan A, Hegazy WAH, Hassan AA (2022). Novel in vivo assessment of antimicrobial efficacy of ciprofloxacin-loaded mesoporous silica nanoparticles against *Salmonella typhimurium* infection. Pharmaceuticals.

[b4-jm-2410015] Alkhalil SS (2024). Fluoroquinolone and enoxacin molecules are potential urease inhibitors for treating ureolytic bacterial infections. Mater Express.

[b5-jm-2410015] Almalki AJ, Ibrahim TS, Elhady SS, Darwish KM, Hegazy WAH (2022). Repurposing α-adrenoreceptor blockers as promising anti-virulence agents in gram-negative bacteria. Antibiotics.

[b6-jm-2410015] Alotaibi HF, Alotaibi H, Darwish KM, Khafagy ES, Abu Lila AS (2023). The anti-virulence activities of the antihypertensive drug propranolol in light of its anti-quorum sensing effects against *Pseudomonas aeruginosa* and *Serratia marcescens*. Biomedicines.

[b7-jm-2410015] Andersson DI, Hughes D (2014). Microbiological effects of sublethal levels of antibiotics. Nat Rev Microbiol.

[b8-jm-2410015] Askoura M, Almalki AJ, Lila ASA, Almansour K, Alshammari F (2021). Alteration of *Salmonella enterica* virulence and host pathogenesis through targeting SdiA by using the CRISPR-Cas9 system. Microorganisms.

[b9-jm-2410015] Askoura M, Hegazy WAH (2020). Ciprofloxacin interferes with *Salmonella typhimurium* intracellular survival and host virulence through repression of *Salmonella* pathogenicity island-2 (SPI-2) genes expression. Pathog Dis.

[b10-jm-2410015] Badr-Eldin SM, Aldawsari HM, Ahmed OA, Kotta S, Abualsunun W (2024). Utilization of zein nano-based system for promoting antibiofilm and anti-virulence activities of curcumin against *Pseudomonas aeruginosa*. Nanotechnol Rev.

[b11-jm-2410015] Bendary MM, Ali MA, Abdel Halim AS, Boufahja F, Chaudhary AA (2024). Investigating sulforaphane’s anti-virulence and anti-quorum sensing properties against *Pseudomonas aeruginosa*. Front Pharmacol.

[b12-jm-2410015] Buckle GC, Walker CL, Black RE (2012). Typhoid fever and paratyphoid fever: Systematic review to estimate global morbidity and mortality for 2010. J Glob Health.

[b13-jm-2410015] Cavalu S, Elbaramawi SS, Eissa AG, Radwan MF, Ibrahim TS (2022). Characterization of the anti-biofilm and anti-quorum sensing activities of the β-adrenoreceptor antagonist atenolol against gram-negative bacterial pathogens. Int J Mol Sci.

[b14-jm-2410015] Charles PG (2017).

[b15-jm-2410015] Cornelis GR (2006). The type III secretion injectisome. Nat Rev Microbiol.

[b16-jm-2410015] Crump JA, Sjolund-Karlsson M, Gordon MA, Parry CM (2015). Epidemiology, clinical presentation, laboratory diagnosis, antimicrobial resistance, and antimicrobial management of invasive *Salmonella* infections. Clin Microbiol Rev.

[b17-jm-2410015] Deiwick J, Nikolaus T, Erdogan S, Hensel M (1999). Environmental regulation of *Salmonella* pathogenicity island 2 gene expression. Mol Microbiol.

[b18-jm-2410015] Easmon CS, Crane JP, Blowers A (1986). Effect of ciprofloxacin on intracellular organisms: In-vitro and in-vivo studies. J Antimicrob Chemother.

[b19-jm-2410015] Elfaky MA, Elbaramawi SS, Eissa AG, Ibrahim TS, Khafagy ES (2023). Drug repositioning: Doxazosin attenuates the virulence factors and biofilm formation in gram-negative bacteria. Appl Microbiol Biotechnol.

[b20-jm-2410015] Elfaky MA, Okairy HM, Abdallah HM, Koshak AE, Mohamed GA (2024). Assessing the antibacterial potential of 6-gingerol: Combined experimental and computational approaches. Saudi Pharm J.

[b21-jm-2410015] Elfaky MA, Thabit AK, Eljaaly K, Zawawi A, Abdelkhalek AS (2022). Controlling of bacterial virulence: Evaluation of anti-virulence activities of prazosin against *Salmonella enterica*. Antibiotics (Basel).

[b22-jm-2410015] Gerlach RG, Hensel M (2007). *Salmonella* pathogenicity islands in host specificity, host pathogen-interactions and antibiotics resistance of *Salmonella enterica*. Berl. Munch. Tierarztl. Wochenschr.

[b23-jm-2410015] Gutiérrez-Castrellón P, Díaz-García L, de Colsa-Ranero A, Cuevas-Alpuche J, Jiménez-Escobar I (2015). Efficacy and safety of ciprofloxacin treatment in urinary tract infections (UTIs) in adults: A systematic review with meta-analysis. Gac Med Mex.

[b24-jm-2410015] Harish BN, Menezes GA (2011). Antimicrobial resistance in typhoidal salmonellae. Indian J. Med. Microbiol.

[b25-jm-2410015] Hegazy WA, Hensel M (2012). *Salmonella enterica* as a vaccine carrier. Future Microbiol.

[b26-jm-2410015] Hegazy WA, Xu X, Metelitsa L, Hensel M (2012). Evaluation of *Salmonella enterica* type III secretion system effector proteins as carriers for heterologous vaccine antigens. Infect Immun.

[b27-jm-2410015] Hegazy WAH (2015). Hepatitis C virus pathogenesis: Serum IL-33 level indicates liver damage. Afr. J. Microbiol. Res.

[b28-jm-2410015] Hegazy WAH, Abbas HA (2017). Evaluation of the role of SsaV ‘*Salmonella* pathogenicity island-2 dependent type III secretion system components on the virulence behavior of *Salmonella enterica* serovar Typhimurium. Afr J Biotechnol.

[b29-jm-2410015] Hegazy WAH, Salem IM, Alotaibi HF, Khafagy ES, Ibrahim D (2022). Terazosin interferes with quorum sensing and type III secretion system and diminishes the bacterial espionage to mitigate the *Salmonella typhimurium* pathogenesis. Antibiotics.

[b30-jm-2410015] Heggie A, Cerny O, Holden DW (2021). STEC and the intracellular *Salmonella*-induced F-actin meshwork. Cell Microbiol.

[b31-jm-2410015] Holzer SU, Hensel M (2012). Divergent roles of *Salmonella* pathogenicity island 2 and metabolic traits during interaction of *S. enterica* serovar Typhimurium with host cells. PLoS One.

[b32-jm-2410015] Hung YT, Lay CJ, Wang CL, Koo M (2017). Characteristics of nontyphoidal *Salmonella* gastroenteritis in Taiwanese children: A 9-year period retrospective medical record review. J Infect Public Health.

[b33-jm-2410015] Hussen NHA, Qadir SH, Rahman HS, Hamalaw YY, Kareem PSS (2023). Long-term toxicity of fluoroquinolones: A comprehensive review. Drug Chem Toxicol.

[b34-jm-2410015] Jałbrzykowska K, Chrzanowska A, Roszkowski P, Struga M (2022). The new face of a well-known antibiotic: A review of the anticancer activity of enoxacin and its derivatives. Cancers.

[b35-jm-2410015] Kamaruzzaman NF, Kendall S, Good L (2017). Targeting the hard to reach: Challenges and novel strategies in the treatment of intracellular bacterial infections. Br J Pharmacol.

[b36-jm-2410015] Khayat MT, Abbas HA, Ibrahim TS, Elbaramawi SS, Khayyat AN (2023). Synergistic benefits: Exploring the anti-virulence effects of metformin/vildagliptin antidiabetic combination against *Pseudomonas aeruginosa* via controlling quorum sensing systems. Biomedicines.

[b37-jm-2410015] Khayat MT, Elbaramawi SS, Nazeih SI, Safo MK, Khafagy ES (2023). Diminishing the pathogenesis of the food-borne pathogen *Serratia marcescens* by low doses of sodium citrate. Biology.

[b38-jm-2410015] Khayat MT, Ibrahim TS, Darwish KM, Khayyat AN, Alharbi M (2022). Hiring of the anti-quorum sensing activities of hypoglycemic agent linagliptin to alleviate the *Pseudomonas aeruginosa* pathogenesis. Microorganisms.

[b39-jm-2410015] Khayat MT, Ibrahim TS, Khayyat AN, Alharbi M, Shaldam MA (2022). Sodium citrate alleviates virulence in *Pseudomonas aeruginosa*. Microorganisms.

[b40-jm-2410015] Knodler LA, Celli J, Hardt WD, Vallance BA, Yip C (2002). *Salmonella* effectors within a single pathogenicity island are differentially expressed and translocated by separate type III secretion systems. Mol Microbiol.

[b41-jm-2410015] Knodler LA, Vallance BA, Hensel M, Jäckel D, Finlay BB (2003). *Salmonella* type III effectors PipB and PipB2 are targeted to detergent-resistant microdomains on internal host cell membranes. Mol Microbiol.

[b42-jm-2410015] Knuff K, Finlay BB (2017). What the SIF is happening? The role of intracellular *Salmonella*-induced filaments. Front Cell Infect Microbiol.

[b43-jm-2410015] Koshak AE, Okairy HM, Elfaky MA, Abdallah HM, Mohamed GA (2024). Antimicrobial and anti-virulence activities of 4-shogaol from grains of paradise against gram-negative bacteria: Integration of experimental and computational methods. J Ethnopharmacol.

[b44-jm-2410015] Kuhle V, Hensel M (2004). Cellular microbiology of intracellular *Salmonella enterica*: Functions of the type III secretion system encoded by *Salmonella* pathogenicity island 2. Cell Mol Life Sci.

[b45-jm-2410015] Kwak YG, Choi SH, Kim T, Park SY, Seo SH (2017). Clinical guidelines for the antibiotic treatment for community-acquired skin and soft tissue infection. Infect Chemother.

[b46-jm-2410015] Lila ASA, Rajab AA, Abdallah MH, Rizvi SMD, Moin A (2023). Biofilm lifestyle in recurrent urinary tract infections. Life.

[b47-jm-2410015] Linares JF, Gustafsson I, Baquero F, Martinez J (2006). Antibiotics as intermicrobial signaling agents instead of weapons. Proc Natl Acad Sci USA.

[b48-jm-2410015] Liu X, Qu X, Wu C, Zhai Z, Tian B (2014). The effect of enoxacin on osteoclastogenesis and reduction of titanium particle-induced osteolysis via suppression of JNK signaling pathway. Biomaterials.

[b49-jm-2410015] Marcus SL, Brumell JH, Pfeifer CG, Finlay BB (2000). *Salmonella* pathogenicity islands: Big virulence in small packages. Microbes Infect.

[b50-jm-2410015] Molina-Quiroz R, Silva C, Molina C, Leiva L, Reyes-Cerpa S (2015). Exposure to sub-inhibitory concentrations of cefotaxime enhances the systemic colonization of *Salmonella typhimurium* in BALB/c mice. Open Biol.

[b51-jm-2410015] Nazeih SI, Ali MA, Halim ASA, Al-Lawati H, Abbas HA (2023). Relocating glyceryl trinitrate as an anti-virulence agent against *Pseudomonas aeruginosa* and *Serratia marcescens*: Insights from molecular and *in vivo* investigations. Microorganisms.

[b52-jm-2410015] Patel JC, Galan JE (2005). Manipulation of the host actin cytoskeleton by *Salmonella*—all in the name of entry. Curr Opin Microbiol.

[b53-jm-2410015] Poh J, Odendall C, Spanos A, Boyle C, Liu M (2008). SteC is a *Salmonella* kinase required for SPI-2-dependent F-actin remodelling. Cell Microbiol.

[b54-jm-2410015] Rahman BA, Wasfy MO, Maksoud MA, Hanna N, Dueger E (2014). Multi-drug resistance and reduced susceptibility to ciprofloxacin among *Salmonella enterica* serovar Typhi isolates from the Middle East and Central Asia. New Microbes New Infect.

[b55-jm-2410015] Rajab AA, Hegazy WA (2023). What’s old is new again: Insights into diabetic foot microbiome. World J Diabetes.

[b56-jm-2410015] Redgrave LS, Sutton SB, Webber MA, Piddock LJV (2014). Fluoroquinolone resistance: mechanisms, impact on bacteria, and role in evolutionary success. Trends Microbiol.

[b57-jm-2410015] Schmidt H, Hensel M (2004). Pathogenicity islands in bacterial pathogenesis. Clin Microbiol Rev.

[b58-jm-2410015] Shaw A, Gullerova M (2021). Home and away: The role of non-coding RNA in intracellular and intercellular DNA damage response. Genes.

[b59-jm-2410015] Stepanovic S, Vukovic D, Dakic I, Savic B, Svabic-Vlahovic M (2000). A modified microtiter-plate test for quantification of staphylococcal biofilm formation. J Microbiol Methods.

[b60-jm-2410015] Tadesse G, Tessema TS, Beyene G, Aseffa A (2018). Molecular epidemiology of fluoroquinolone resistant *Salmonella* in Africa: A systematic review and meta-analysis. PLoS One.

[b61-jm-2410015] Thabit AK, Eljaaly K, Zawawi A, Ibrahim TS, Eissa AG (2022a). Muting bacterial communication: Evaluation of prazosin anti-quorum sensing activities against gram-negative bacteria *Pseudomonas aeruginosa*, *Proteus mirabilis*, and *Serratia marcescens*. Biology (Basel).

[b62-jm-2410015] Thabit AK, Eljaaly K, Zawawi A, Ibrahim TS, Eissa AG (2022b). Silencing of *Salmonella typhimurium* pathogenesis: Atenolol acquires efficient anti-virulence activities. Microorganisms.

[b63-jm-2410015] Troman LA, Collinson I (2021). Pushing the envelope: The mysterious journey through the bacterial secretory machinery, and beyond. Front Microbiol.

[b64-jm-2410015] Uche IV, MacLennan CA, Saul A (2017). A systematic review of the incidence, risk factors and case fatality rates of invasive nontyphoidal *Salmonella* (iNTS) disease in Africa (1966 to 2014). PLoS Negl Trop Dis.

[b65-jm-2410015] Vernikos GS, Parkhill J (2006). Interpolated variable order motifs for identification of horizontally acquired DNA: Revisiting the *Salmonella* pathogenicity islands. Bioinformatics.

[b66-jm-2410015] Vesterlund S, Paltta J, Karp M, Ouwehand AC (2005). Measurement of bacterial adhesion—In vitro evaluation of different methods. J Microbiol Methods.

[b67-jm-2410015] Wain J, Hendriksen RS, Mikoleit ML, Keddy KH, Ochiai RL (2015). Typhoid fever. Lancet.

[b68-jm-2410015] Xu X, Hegazy WA, Guo L, Gao X, Courtney AN (2014). Effective cancer vaccine platform based on attenuated *Salmonella* and a type III secretion system. Cancer Res.

[b69-jm-2410015] Yao C, Zhu M, Han X, Xu Q, Dai M (2021). A bone-targeting enoxacin delivery system to eradicate *Staphylococcus aureus*-related implantation infections and bone loss. Front Bioeng Biotechnol.

[b70-jm-2410015] Yin J, Chen Y, Xie X, Xia J, Li Q (2017). Influence of *Salmonella enterica* serovar Pullorum pathogenicity island 2 on type III secretion system effector gene expression in chicken macrophage HD11 cells. Avian Pathol.

[b71-jm-2410015] Yu XJ, Grabe GJ, Liu M, Mota LJ, Holden DW (2018). SsaV interacts with SsaL to control the translocon-to-effector switch in the *Salmonella* SPI-2 type three secretion system. MBio.

[b72-jm-2410015] Zehra F, Fatima SM, Syedain F (2015). Sensitivity pattern of *Salmonella* species in different age groups. Int J Endors Health Sci Res.

